# The interaction between Septoria stem canker and the mycobiome of *Populus trichocarpa* stems

**DOI:** 10.1128/msystems.00055-26

**Published:** 2026-06-15

**Authors:** Morgan G. A. Miller, Gillian E. Bergmann, Ricardo I. Alcalá Briseño, Yung-Hsiang Lan, Kelsey L. Søndreli, Posy E. Busby, Jared M. LeBoldus

**Affiliations:** 1 Department of Botany and Plant Pathology, Oregon State University, Corvallis, Oregon, USA; 2 Forest Engineering, Resources and Management Department, Oregon State University, Corvallis, Oregon, USA; Boise State University, Boise, Idaho, USA

**Keywords:** microbiome, microbial invasion, *Sphaerulina musiva*, Septoria canker, poplar, phytobiome, microbial ecology

## Abstract

*Sphaerulina musiva*, a fungal pathogen causing leaf spot and canker disease of poplar trees (*Populus* spp.), was recently introduced to the Pacific Northwest, where it threatens commercially valuable plantations and native riparian ecosystems. Vascular tissue was collected from the stems of 410 symptomatic and asymptomatic trees in an *S. musiva*-infested *Populus trichocarpa* plantation. Resident fungal endophyte communities were characterized with ITS amplicon sequencing. Canker expression and *S. musiva* presence corresponded with reduced fungal diversity across multiple indices. Fungal endophyte communities of symptomatic tissues were frequently dominated by *S. musiva* and thus compositionally distinct from those of asymptomatic tissues. Asymptomatic-tissue communities from healthy and diseased stems did not differ in composition or diversity, indicating disease-associated low-diversity mycobiome states are local to the cankered site. The relative abundance of *S. musiva* was positively correlated with stem cankering. *S. musiva* was negatively correlated with key non-pathogenic fungal endophytes, which were themselves strongly inter-correlated. Our results illustrate *S. musiva*’s ability to exploit the vascular microhabitat of susceptible *Populus trichocarpa* stems and dominate resident fungal assemblies at the site of infection.

**IMPORTANCE:**

Plant-associated microbial communities can both mediate and be modified by pathogen infection. Thus, understanding disease outcomes of complex plant pathosystems requires characterization of pathogen-phytobiome interactions. Efforts to characterize these interactions have yielded microecological insights with applied relevance for disease management in herbaceous leaf- and root-associated pathosystems. However, pathogen-phytobiome interactions in the vascular tissues of hardwood stems remain largely unexplored. Our findings illuminate the ecological organization of the *Populus trichocarpa* stem mycobiome under *S. musiva* disease pressure and advance understanding of microfungal community dynamics in the Septoria stem canker pathosystem. Additionally, we identify potentially interactive fungal taxa that may disproportionately shape mycobiome structure and disease dynamics in *Populus trichocarpa* stems.

## INTRODUCTION

Communities of non-pathogenic microbes living in plant tissues (endophytes) can modify disease expression in their hosts (1–4). Complex interactions between pathogenic and non-pathogenic members of the phytobiome, environmental factors, and host genetic resistance collectively contribute to disease severity (5–7). As diverse and dynamic ecological systems, endophyte communities may influence disease processes through interactions between individual taxa and at the community level (8, 9). Reciprocally, plant pathogens may directly or indirectly manipulate resident endophyte communities to facilitate infection (10–12). Characterization of complex pathogen–phytobiome relationships will be essential for improved prediction of disease outcomes across pathosystems and plant compartments (13–16).

Fungal endophytes of plant stems remain relatively understudied compared with those of leaf and root endospheres (17, 18). The unique spatial constraints, biochemical and nutrient profile, and highly protected nature of vascular stem tissues regulate fungal colonization, biomass, and ecological function (19–22). Consequently, niche-specific microbial occupancy and resource utilization patterns may uniquely mediate pathogen invasion and disease progression in plant stems (23). Pathogenic exploitation of vascular stem tissues may reciprocally disrupt their normal microecology (11, 24). Analogous two-way dynamics are well documented in a variety of herbaceous leaf- and root-associated pathosystems. For example, *Rhizoctonia solani* invasion of sugar beet roots alters fungal and bacterial endophyte composition while inducing disease-suppressive metabolic shifts within bacterial communities (25). *Fusarium* diseases similarly reshape microbial community structure and disease-modification potential in below- and aboveground tissues of chili pepper, bean, and tomato (26–28). Analogous disease response processes may also occur in the stem phytobiome of hardwood species, but these dynamics remain understudied.

Poplars (*Populus* spp.) are hardwoods with global distributions, ecological importance, and commercial value for fiber and biofuel production (29). Fungal pathogens of *Populus* limit plantation productivity and threaten naive poplar populations (30). *Sphaerulina musiva* (syn. *Septoria musiva*) causes Septoria leaf spot and stem canker disease in susceptible *Populus* species. *S. musiva* infects *Populus* in a polycyclic manner. Wind and rain splash disperse sexual ascospores in spring to initiate long-distance primary infection. Asexual conidia production in summer promotes repeated secondary infection of local hosts. As a hemibiotroph, *S. musiva* establishes biotrophic infection before transitioning to necrotrophy, whereafter host tissues are degraded and killed to acquire nutrients (31). Necrotic leaf spots form around infected stomata, which reduce the photosynthetic capacity of the host and can cause premature defoliation. Vascular tissues are invaded and killed during stem infection, producing necrotic cankers that expand as the disease progresses (32–34). Severe cankering deforms, girdles, and weakens stems, increasing the risk of breakage or killing the tree outright (35). The early 21st-century anthropogenic introduction of *S. musiva* to the Pacific Northwest motivates research and management efforts to protect planted and natural stands of highly susceptible *P. trichocarpa* and hybrid *Populus* (36–38).

*S. musiva* is adapted to exploit the *Populus* stem microhabitat (22, 34, 39, 40). Disturbances associated with *S. musiva* establishment likely correspond with community-level microbial shifts, potentially resembling dynamics observed in citrus, oak, pine, and chestnut pathosystems (41–44). However, the mechanisms governing phytobiome response in the *S. musiva-Populus* pathosystem, particularly within vascular stem tissues, are not well characterized. While studies of similar hardwood pathosystems are informative, species-specific biochemical interactions may uniquely shape phytobiome disease response in the *S. musiva-Populus* pathosystem.

Direct endophyte-pathogen interaction and microhabitat modification at the site of infection can drive local endophyte selection (10, 32). Chemical antagonism and competitive exclusion of resident fungal endophytes during biotrophic *S. musiva* infection may thus alter fungal community composition prior to canker development (12, 30, 45, 46). Cell wall digestion and tissue necrosis during canker development may promote saprotrophic fungal endophytes, resulting in trophic structure disruption or transition as symptoms progress (47). Systemic immune-related metabolic shifts can shape biochemical endophyte selection across symptomatic and asymptomatic host tissues (17, 48, 49). A diverse arsenal of effectors secreted by *S. musiva* are known to disrupt *Populus* immune processes (40). Effector-induced immune disruption may facilitate secondary infection or alter endophyte selection in tissues distant from the site of primary infection (50).

Reciprocally, resident endophyte communities of *Populus* stems may influence *S. musiva* infection and canker development. Multiple studies have illuminated microbial disease modification effects in *Populus* pathosystems, including bacterial biocontrol of *S. musiva* (30, 51–56). For example, a taxonomically diverse group of foliar fungal endophytes has been shown to modify *Melampsora* rust severity across *Populus* genotypes, either increasing or decreasing disease severity of local tissues in greenhouse inoculation experiments (57). Landscape-scale associations between foliar mycobiome composition and *Melampsora* rust severity of wild *P. trichocarpa* further suggest community-level disease modification processes may be epidemiologically important (58). The *Populus* stem mycobiome could possess similar disease modification potential. Fungal endophytes of *Populus* stems may physically or chemically inhibit *S. musiva* colonization through host immune priming, direct chemical antagonism, niche preemption, or microhabitat modification (1, 4, 5, 13, 59, 60). Once *S. musiva* becomes established, fungal endophytes may continue to modify disease expression via host immune modulation, hyperparasitism, or opportunistic pathogenicity.

The invasion resistance of synthetic and *in planta* microbial communities depends on community-level characteristics, including biodiversity, trophic organization, and ecological complexity (61, 62). For example, simplified bacterial communities of maize roots inhibit *Fusarium verticillioides* invasion and disease expression more effectively than any single constituent taxa (63). High trophic connectivity and niche occupancy within rhizosphere bacterial communities constrain *Ralstonia solanacearum* invasion and disease expression in tomato, highlighting community-level resource coverage as a key barrier for pathogen invasion and disease progression (64). Understanding the diversity and ecological structure of the *Populus* stems mycobiome will illuminate how pre-invasion fungal composition could either preclude or facilitate *S. musiva* invasion and disease progression (65). Differential invasion resistance resulting from spatial or genotype-associated stem mycobiome composition variation may shape *S. musiva* epidemiology at local and broad scales (7, 61, 66–68).

To better understand the relationship between Septoria stem canker disease and the *Populus* stem mycobiome, we conducted a fungal metabarcoding study of an *S. musiva* infested *P. trichocarpa* common garden. Three primary research objectives were to (i) characterize the association between Septoria stem canker expression and the diversity and composition of fungal endophyte communities, (ii) assess whether this association is systemic throughout the diseased stem or local to the site of infection, and (iii) identify fungal endophytes strongly associated with *S. musiva* and canker expression. We tested three related hypotheses: (i) fungal endophyte communities of symptomatic stem tissues will be less diverse and compositionally distinct compared with those of asymptomatic stem tissues; (ii) fungal endophyte communities of asymptomatic tissues of cankered stems will be less diverse and compositionally distinct compared with those of entirely healthy stems; and (iii) common fungal endophytes will be negatively correlated with relative *S. musiva* abundance and canker expression. We predict that compositional differences between symptomatic and asymptomatic tissues will be largely attributable to increased taxonomic turnover, consistent with tissue degradation facilitating colonization by opportunistic decay- or disease-associated fungi or with pre-infection endophyte compositions predisposing stem tissues to *S. musiva* infection. We predict that compositional differences between asymptomatic tissues of cankered stems and those of entirely healthy stems will reflect reduced taxonomic turnover, consistent with systemic host-mediated physiological changes associated with infection stress or immune response that modulate the relative abundance of resident endophytes, potentially resulting in loss of resident taxa under strong selection. Overall, our study characterizes the understudied vascular stem mycobiome of *P. trichocarpa* and illuminates community-level associations between this system and Septoria canker expression.

## MATERIALS AND METHODS

### Common garden sampling

In 2018, *Sphaerulina musiva* was isolated from cankered *Populus trichocarpa* in a mature common garden plantation in Boardman, Morrow County, Oregon, United States (69). A total of 508 woody tissue samples were collected at breast height (approximately 1.37 m) from 410 randomly selected symptomatic and asymptomatic trees (one selection per genotype). A single healthy tissue sample was collected from each asymptomatic tree. Two samples were collected from each symptomatic tree. Cankered-tissue samples were collected from the margin separating necrotic and healthy tissue. Non-cankered tissue samples were collected from completely healthy regions of living wood approximately 15 cm below the cankered tissue sample location. A sanitized chisel was used to extract a 5 cm^2^ piece of bark, vascular tissue, and sapwood from each sample location. Samples were kept on ice during transport and subsequently stored at −80°C. Tissue disease status (classified as healthy, cankered, or non-cankered) reflects the disease phenotype observed during sampling and was used for hypothesis testing.

### Extraction and sequencing

Approximately 40 mg of vascular tissue (including xylem, vascular cambium, and phloem) was collected from each sample using sterile tools. Vascular tissue was lyophilized for 72 h, and DNA was extracted using a 96-well SYNERGY Plant DNA Extraction Kit (OPS Diagnostics LLC, New Jersey, USA). DNA product was purified using Agencourt AMPure XP beads (1:1 DNA to bead ratio, Beckman-Coulter Inc., California, USA). DNA quantification and quality assessment were conducted with Nanodrop and Qubit (Thermo Fisher Scientific, Massachusetts, USA). Amplicon libraries were prepared following a two-stage amplification protocol. ITS3_KYO1 and ITS4 primers (modified with adaptors and 3–6 bp heterogeneous spacers) were chosen to target the ITS2 region (70, 71). Stage-one PCRs contained 12.5 µL of MyFi Master Mix (Bioline, Tennessee, USA), 1.25 µL of each primer, 2 µL of template DNA, 5.5 µL of sterile water, and 2.5 µL of plant-specific peptide nucleotide acid (PNA) clamp IP01 (PNA Bio, California, USA) to reduce host amplification. PCR cycling conditions were: 95℃ for 3 min, 32 cycles of 95℃ (30 s), 78℃ (5 s), 50℃ (30 s), and 72℃ (30 s), and a final extension stage at 72℃ for 3 min. Stage-two PCRs contained 12.5 µL of MyFi Master Mix, 2 µL of amplicon solution, 2.5 µL of Illumina adapter mixture, and 8 µL of sterile water (72). PCR cycling conditions were: 95℃ for 1 min, 8 cycles of 95℃ (20 s), 55℃ (20 s), and 72℃ (40 s), and a final extension stage at 72℃ for 5 min. Gel electrophoresis was used to verify PCR product size and assess potential contamination in extraction blanks and PCR negative controls. PCR product was purified and normalized using a Just-a-Plate purification kit (Charm Biotech, Missouri, USA). Equimolar concentrations of each sample were pooled before sequencing (Illumina MiSeq, sample-multiplexed, 300-bp paired-end, Center for Genome Research and Biocomputing, Oregon State University).

### Data processing

Data processing, analysis, and visualization were conducted primarily in R v. 4.5.1 (73–78). Raw sequence data were demultiplexed with Pheniqs v. 1.0 (maximum likelihood threshold = 0.995) (79). Read count and quality statistics were assessed with SeqKit v. 2.11.0 (80). Quality filtering was conducted with DADA2 v. 1.36.0 (81) following an adapted procedure from the ITS workflow documentation: (i) reads containing ambiguous base calls were removed, (ii) primer sequences were trimmed with cutadapt v. 4.2 (82), (iii) base calls following a Phred Quality Score of 28 (99.84% confidence) were trimmed, and (iv) short reads (fewer than 90 nucleotides) were removed. Standard amplicon sequence variant (ASV) inference (error rate learning, dereplication, de-noising, paired read merging, and chimera removal) was conducted according to DADA2 documentation. Taxonomic assignment was conducted using DADA2’s Naïve Bayesian Classifier and the UNITE general FASTA release for eukaryotes database (83–85). As *S. musiva* was absent from the UNITE database, the pathogen’s ITS2 sequence (GenBank accession: MN275180.1) was retrieved and added to the database before taxonomic assignment. ASVs appearing in control samples at proportions exceeding 10% of their total read counts were classified as putative contaminants and removed from all samples (19 ASVs, representing 1.1% of the total read count of the sequence table). ASVs classified as *Viridiplantae* were removed from all samples (241 ASVs, representing 73.3% of the total read count of the sequence table). ASVs classified as non-fungal eukaryotes or with unresolved taxonomy were also removed from all samples (15 ASVs, representing 0.1% of the total read count of the sequence table). Samples containing fewer than 1,000 fungal reads were discarded (322 samples, 63.4% of all samples). The high abundance of *Viridiplantae* reads suggests that the plant-specific PNA clamps did not fully suppress host amplification, resulting in reduced fungal sequencing depth in many samples and substantial data loss during filtering.

### Diversity analysis

Diversity analyses were conducted with vegan v. 2.7.2 (86). Species richness, Shannon–Weaver, Simpson, and Inverse Simpson indices were selected to quantify community alpha diversity. Pielou’s evenness and Berger–Parker dominance indices were selected to quantify community evenness and single-ASV dominance, respectively. Aitchison distance was selected to quantify beta dissimilarity while mitigating compositional bias (pseudo-count = 1). Repeated empirical sequence count rarefaction was implemented before both alpha- and beta-diversity analyses to control sequence-depth bias (87). Mean alpha diversity and pairwise beta dissimilarity values were calculated after 50,000 rarefaction repetitions to a depth of 1,000 reads. Statistical comparisons were performed only between healthy and symptomatic groups (i.e., healthy–cankered and healthy–non-cankered), as paired symptomatic groups were statistically dependent. Welch’s two-sample *t*-tests were used to compare the mean alpha diversity of independent disease status groups. Permutational multivariate analysis of variance (PERMANOVA) and permutational multivariate analysis of dispersion (PERMDISP) tests were used to compare community composition and dispersion between independent disease status groups (permutations = 1,000). Nonmetric multi-dimensional scaling was used to ordinate Aitchison distances for visualization. Total-sum-scaled sequence counts aggregated by taxon were treated as continuous variables, fitted as linear trend surfaces onto the ordination, and visualized as vectors representing gradients of increasing relative abundance. Pairwise Jaccard dissimilarity among fungal communities was partitioned into turnover (defined by few shared taxa and many taxa unique to each community) and nestedness (the complement of turnover) components with betapart v. 1.6.1 and summarized for all within- and between-disease status comparisons (88, 89).

### Correlation analysis

Pairwise ASV-ASV correlations were estimated with the sparse correlations for compositional data (SparCC) algorithm as implemented in FastSpar v. 1.0.0 (90, 91). The significance of ASV-ASV correlations was evaluated using SparCC’s bootstrap approach (iterations = 20,000). Point-biserial correlation was used to quantify ASV-phenotype association after total sum scaling. Pearson correlation coefficients (estimated by SparCC) and point-biserial correlation coefficients are hereafter denoted as *r*_cc_ and *r*_pb_, respectively. Only healthy- and cankered-tissue community data were used for correlation network inference. Weak (|*r*| < 0.3) or statistically insignificant (*P*-adj. > 0.05, Bonferroni) correlations were assumed to be uninformative or indirect and were removed before correlation network construction. ASV-ASV and ASV-phenotype networks were trimmed, joined, and visualized with igraph v. 2.2.1 (92, 93).

## RESULTS

### Fungal community composition

**Fig 1:**
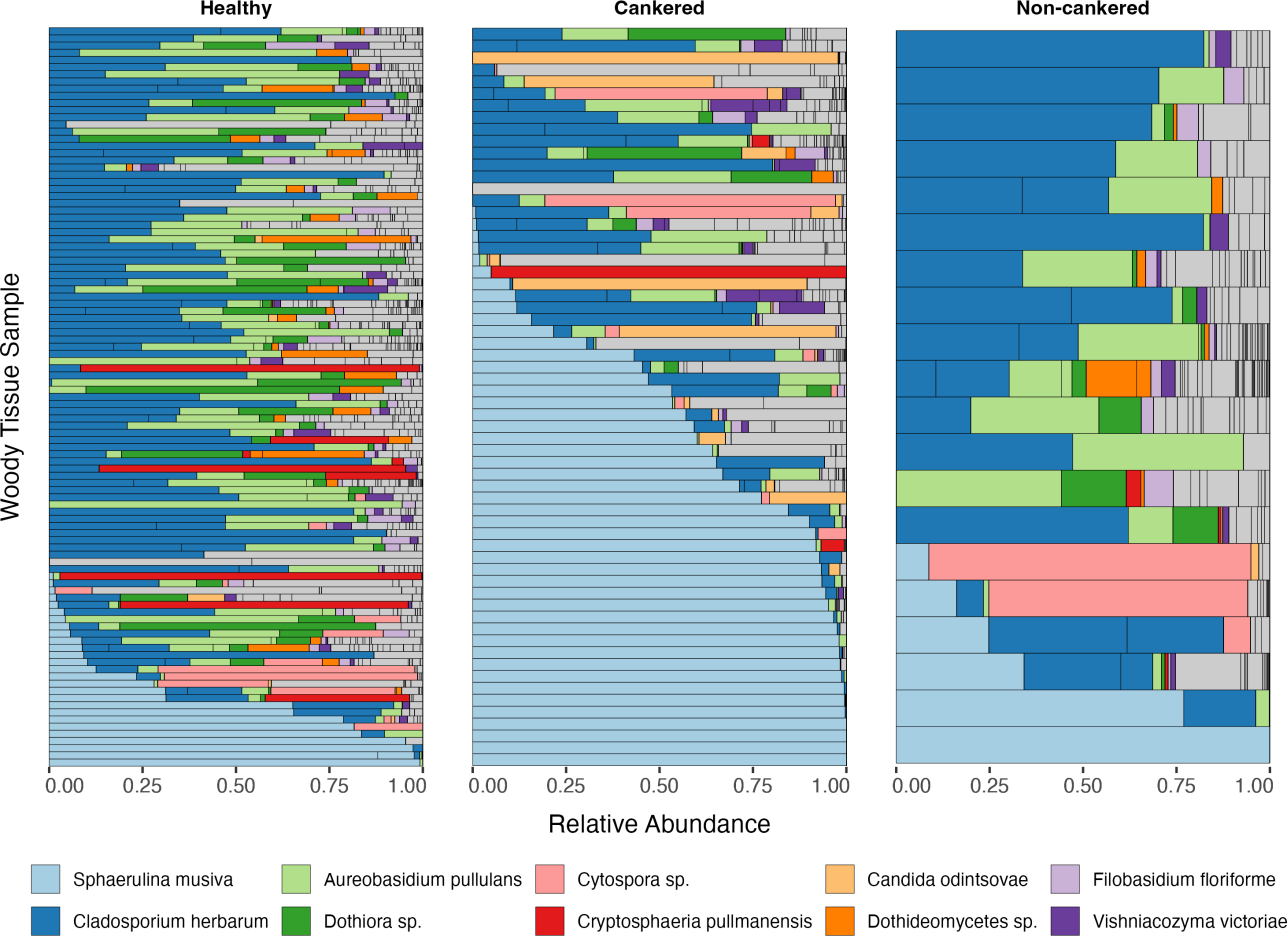
A bar chart depicting per-sample fungal endophyte community composition by disease status: healthy (*n* = 103), cankered (*n* = 62), and non-cankered (*n* = 20). The 10 fungal taxa with the highest mean relative abundance across all samples are colored distinctly. All other fungal taxa are colored in gray.

A total of 306 fungal ASVs and 185 woody tissue samples (103 healthy, 62 cankered, and 20 non-cankered) remained after data processing. Most ASVs occurred rarely. A total of 269 ASVs occurred in fewer than 10 samples. A comparable number of ASVs were classified as Ascomycota and Basidiomycota (174 and 130, respectively). Ascomycota ASVs represented 88.7% of fungal reads. Two ASVs were classified as *S. musiva*, representing 30.2% and 0.1% of fungal reads, respectively. The high-abundance *S. musiva* ASV disproportionately dominated cankered-tissue communities. Fungal endophyte composition was heterogeneous when not dominated by the high-abundance *S. musiva* ASV, with *Cladosporium herbarum* and *Aureobasidium pullulans* ASVs often co-abundant (Fig. 1). ASVs classified as *Dothiora* sp., *Cytospora* sp., and *Cryptosphaeria pullmanensis* were moderately abundant and dominated some communities. *S. musiva* ASVs were present in 27 healthy-tissue communities and absent from 15 cankered-tissue communities (Fig. 1). The relative abundance of *S. musiva* ASVs was significantly predictive of canker expression (*P* < 0.001, binomial regression). While relative dominance of *S. musiva* strongly differentiates mycobiome composition, this pattern may reflect the compositional nature of amplicon sequencing data, whereby proliferation of *S. musiva* can produce apparent relative declines in co-occurring taxa without corresponding reductions in their absolute abundance.

### Relationships between disease expression and fungal diversity

**Fig 2:**
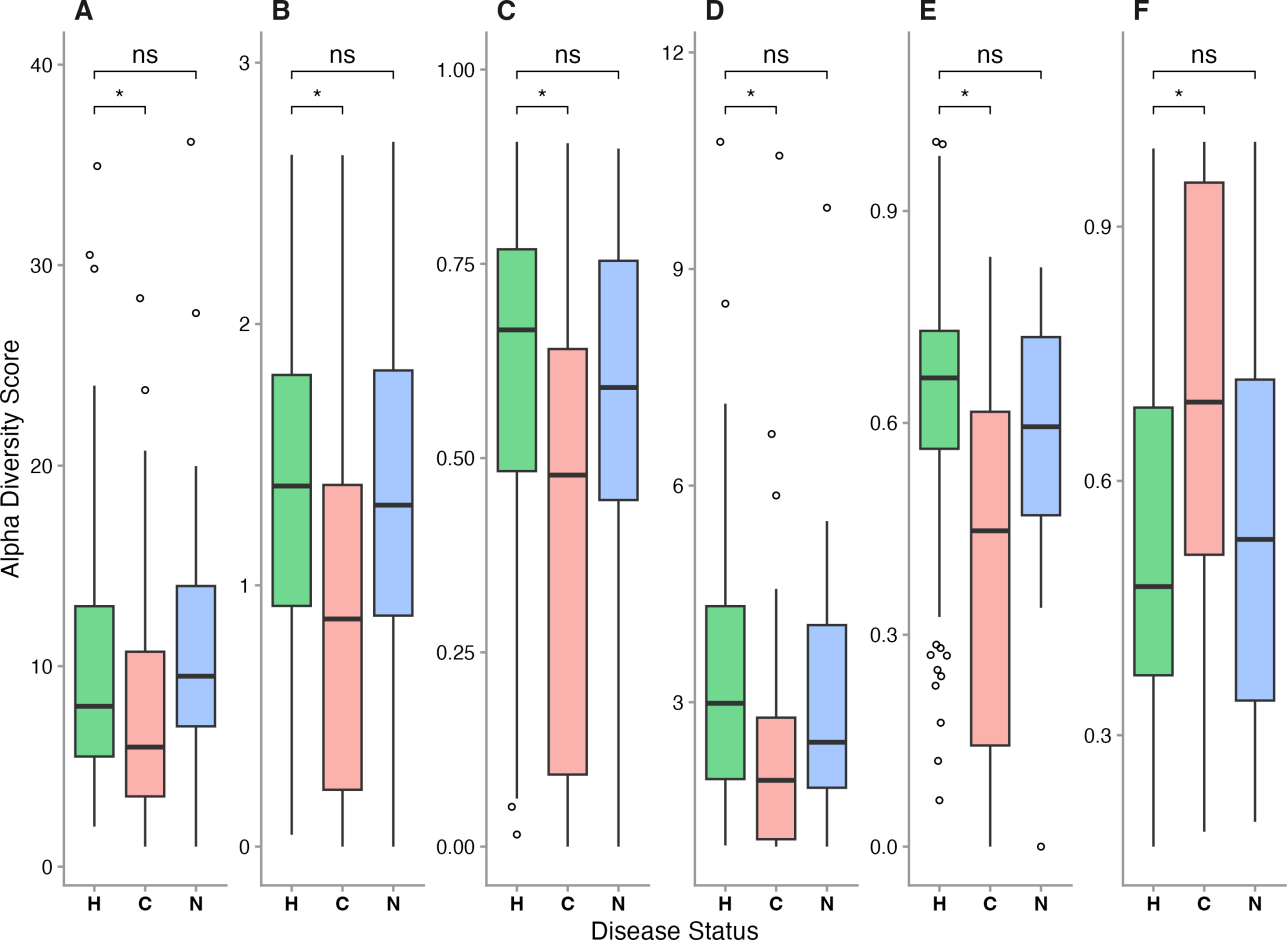
Box and whisker plots depicting fungal endophyte community alpha diversity grouped by disease status: healthy (H; *n* = 103), cankered (C; *n* = 62), and non-cankered (N; *n* = 20). Five different indices were compared: richness (**A**), Shannon-Weaver (**B**), Simpson (**C**), Inverse Simpson (**D**), Pielou evenness (**E**), and Berger-Parker dominance (**F**). Mean diversity scores were calculated for each sample after repeated rarefaction. Asterisks denote statistically significant differences in mean values between independent disease status groups, determined by Welch’s two-sample *t*-tests (*P* < 0.05).

**Fig 3:**
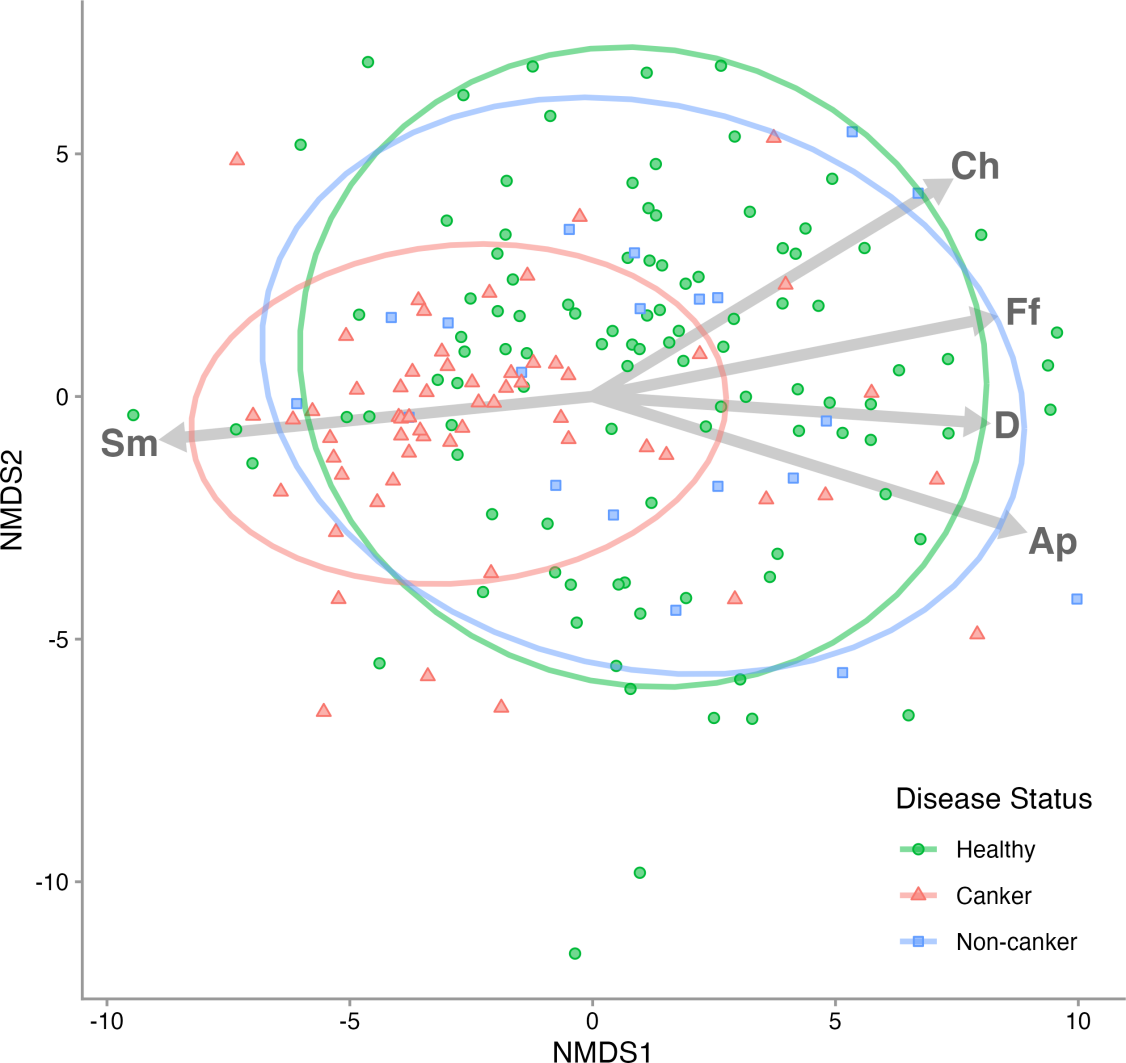
Nonmetric multi-dimensional scaling ordination depicting fungal endophyte community dissimilarity in ASV space (rarefied Aitchison distance) across healthy (*n* = 103), cankered (*n* = 62), and non-cankered (*n* = 20) disease statuses. Healthy and cankered tissue communities differed in composition (PERMANOVA, *P* < 0.001) and dispersion (PERMDISP, *P* < 0.001), whereas no differences were detected between healthy and non-cankered tissue communities (*P* > 0.9, PERMANOVA; *P* > 0.6, PERMDISP). Linear trend surfaces fitted for the five fungal taxa most strongly correlated with the ordination are depicted as vectors representing both the direction and strength of increasing relative abundance: *Aureobasidium pullulans* (Ap, *R*^2^ = 0.290), *Sphaerulina musiva* (Sm, *R*^2^ = 0.265), *Cladosporium herbarum* (Ch, *R*^2^ = 0.247), *Filobasidium floriforme* (Ff, *R*^2^ = 0.238), and *Dioszegia* sp. (D, *R*^2^ = 0.221). Ellipses were drawn assuming multivariate *t*-distributions (confidence level = 95%). Two-dimensional ordination produced a final stress of 0.210.

Healthy-tissue communities were more diverse than cankered-tissue communities (*P* < 0.03, all diversity indices, Welch’s two-sample *t*-test; Fig. 2). Cankered-tissue communities were less even and more frequently dominated by a single ASV than healthy-tissue communities (*P* < 0.00001, Pielou’s evenness and Berger-Parker dominance indices, Welch’s two-sample *t*-test; Fig. 2). The diversity, evenness, and single-ASV dominance of healthy- and non-cankered-tissue communities did not differ significantly (*P* > 0.2, all indices, Welch’s two-sample *t*-test; Fig. 2). *S. musiva* ASV presence was associated with reduced community diversity and evenness and increased single-ASV dominance (*P* < 0.002, all indices, Welch’s two-sample *t*-test; Fig. S1). Healthy and cankered-tissue communities were compositionally distinct (*P* < 0.001, R^2^ = 0.060, pseudo-F_(1, 163)_ = 10.49, PERMANOVA). Cankered-tissue communities were less dispersed in ASV space than healthy-tissue communities [*P* < 0.001, F_(1, 163)_ = 23.30, PERMDISP]. Cankered-tissue communities cluster in ordination space along the gradient of increasing relative *S. musiva* abundance (Fig. 3). No significant differences in composition or dispersion were found between healthy and non-cankered tissue communities (*P* > 0.9, PERMANOVA; *P* > 0.6, PERMDISP). Jaccard dissimilarity was uniformly high within and between disease status groups (medians across all comparisons 0.82–0.86; Fig. S2 panel A). Turnover constituted the majority of Jaccard dissimilarity for all comparisons (medians 0.86–0.92; Fig. S2 panel B). These patterns reflect high presence–absence variation and fungal community heterogeneity independent of disease status.

### ASV-phenotype correlation network

**Fig 4:**
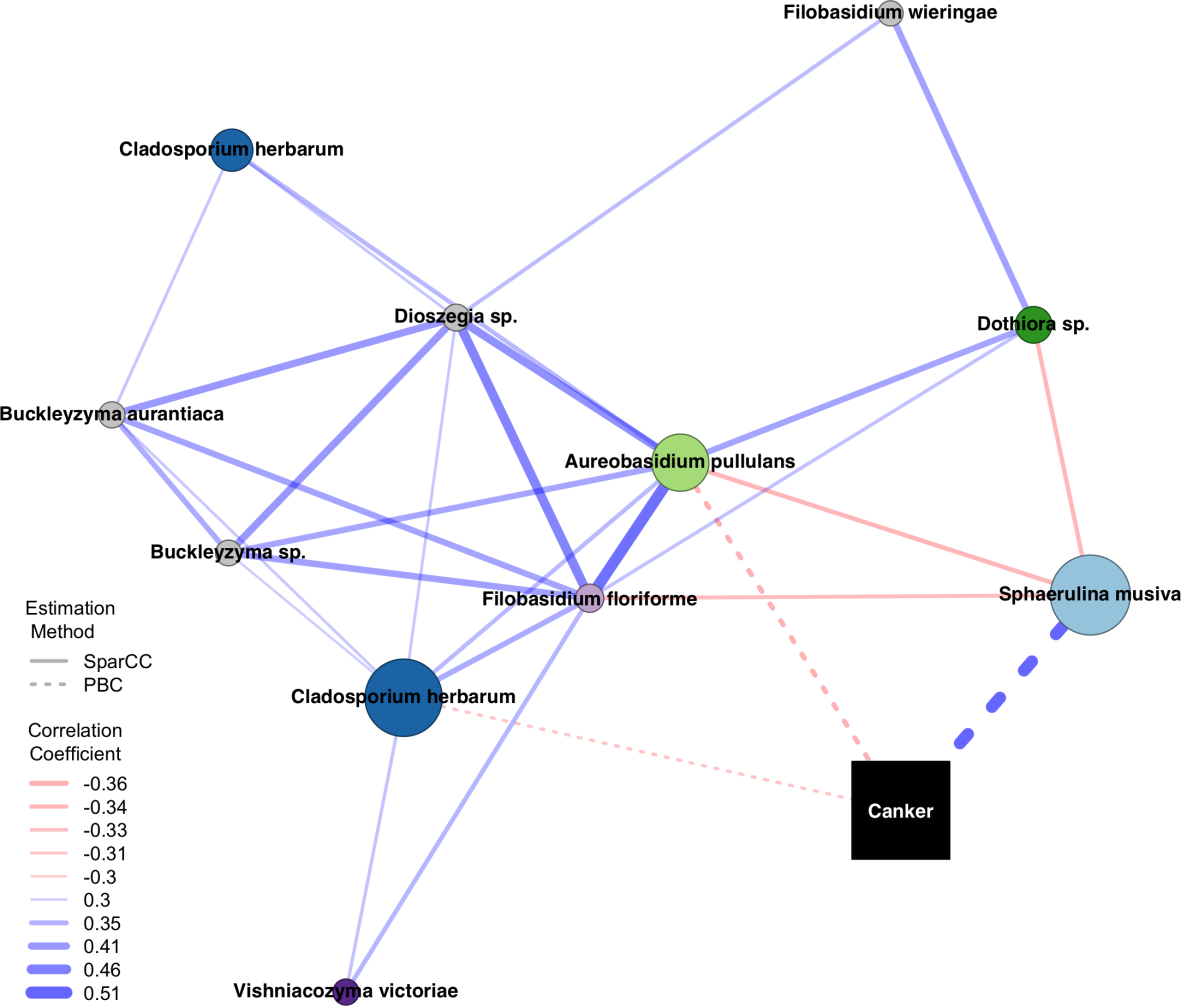
Joined ASV-ASV and ASV-phenotype correlation network of healthy and cankered tissue communities. Circular nodes represent ASVs. A square node represents the canker expression phenotype. Solid and dashed lines represent ASV-ASV (SparCC) and ASV-phenotype (point-biserial correlation) correlations, respectively. ASV node size corresponds to the ASV’s mean relative abundance within healthy- and cankered-tissue communities. ASV nodes are colored according to their mean relative abundance across all samples.

The relative abundance of the high-abundance *S. musiva* ASV was positively correlated with stem cankering (*r_pb_* = 0.511). The relative abundances of an *Aureobasidium pullulans* ASV and a *Cladosporium herbarum* ASV were negatively correlated with stem cankering (*r_pb_* = −0.356 and −0.317 respectively). The high-abundance *S. musiva* ASV was negatively correlated with *Aureobasidium pullulans* (*r_cc_* = −0.346), *Dothiora* sp. (*r_cc_* = −0.341), and *Filobasidium floriforme* (*r_cc_* = −0.334) ASVs. Pairwise correlations among non-*S*. *musiva* ASVs were exclusively positive, with the correlations among ASVs classified as *Aureobasidium pullulans*, *Filobasidium floriforme*, *Dioszegia* sp., *Buckleyzyma aurantiaca*, and *Buckleyzyma* sp., forming a strongly inter-correlated group (*r_cc_* range: 0.391–0.491). Various other ASVs classified as *Cladosporium herbarum*, *Dothiora* sp., *Filobasidium wieringae*, and *Vishniacozyma victoriae* were correlated with these five key ASVs and each other (*r_cc_* range: 0.301–0.387; Fig. 4).

## DISCUSSION

Plant-associated microbiota are increasingly recognized as important components of plant pathosystems (2, 3, 7, 8, 13, 94). A growing body of evidence indicates that phytobiome manipulation is a common capacity of plant pathogens (10, 12). Additionally, endophyte communities can influence phytopathogen invasion and disease progression (5). This reciprocal relationship is mediated by microhabitat, host, and environmental factors, which collectively contribute to the context-dependent nature of endophyte disease ecology (6). Thus, a robust understanding of how the phytobiome responds to and influences disease processes in key plant pathosystems will be essential for accurate modeling, improved forecasting, and informed management of plant disease in natural and agricultural ecosystems (95).

This study characterizes the understudied vascular stem mycobiome of *P. trichocarpa* under *S. musiva* disease pressure and illuminates associations between mycobiome composition and disease expression in the Septoria stem canker pathosystem. Canker expression was associated with reduced mycobiome diversity at the site of infection, but not throughout the symptomatic stem. *S. musiva* disproportionately dominated cankered-tissue communities, reducing fungal diversity and compositionally differentiating symptomatic and asymptomatic mycobiomes. Compositional differentiation of cankered tissue communities was best explained by variation in relative abundance, particularly the dominance of *S. musiva*, rather than consistent patterns of taxonomic turnover, which were uniformly high within and between disease status groups and largely attributable to heterogeneous presence–absence variation among non-dominant taxa. These findings indicate *S. musiva* may directly or indirectly manipulate resident fungal endophyte communities at the site of infection. Alternatively, reduced-diversity pre-infection fungal composition may facilitate *S. musiva* infection and niche exploitation.

### Fungal endophyte communities of *P. trichocarpa* stems in a diseased common garden setting

In addition to biogeographic and host-genotype effects, tissue-specific selection strongly shapes endophyte composition across *Populus* compartments (68, 96–100). Physiological and biochemical characteristics of vascular stem tissues differentiate fungal communities of *Populus* stems from those of leaves and roots (24). Assessment of inferred fungal endophyte morphology may reveal potential selective effects of the vascular stem microhabitat.

Our results support both filamentous and yeast-like microfungi as abundant in the vascular stem tissues of *P. trichocarpa* under *S. musiva* disease pressure. Inferred high-abundance taxa, including *Cladosporium herbarum*, *Cytospora* sp., and *Cryptosphaeria pullmanensis*, exhibit primarily filamentous morphology (101–105). Primarily yeast-like taxa, including *Candida odintsovae*, *Vishniacozyma victoriae*, *Dioszegia* sp., and *Buckleyzyma* sp., were also frequently inferred (106). The dominant form of polymorphous endophytes, including *Aureobasidium pullulans*, *Dothiora* sp., and *Filobasidium* sp., remain unclear in this microhabitat (107, 108).

Tissue-specific physiology, substrate availability, and secondary metabolite composition may favor certain microfungal growth forms in the vascular stems of woody plants. Endophyte-endophyte interactions may additionally promote morphological transition during intravascular colonization. Morphological diversity may shape niche occupancy, resource utilization, and disease dynamics in the *Populus* stem mycobiome, as microfungal morphology considerably modifies an endophyte’s fitness in the stem endosphere (19, 24). For example, filamentous growth enables penetration of the stem epidermis through wounds or lenticels and hyphal traversal of adjacent xylem vessels (22, 23). Additionally, unicellular conidia and yeast-like microfungi can be passively transported by the transpiration stream, which facilitates rapid systemic colonization (3, 109).

Stem cankering generally corresponded with *S. musiva* abundance. However, *both* asymptomatic colonization of *P. trichocarpa* by *S. musiva* and stem cankering in the absence of *S. musiva* were observed. Asymptomatic *S. musiva* colonization is unsurprising given the pathogen’s hemibiotrophic life strategy. Asymptomatic tissues colonized by *S. musiva* likely represent initial biotrophic stages of infection, before necrotrophic transition produces observable cankering. Genotypic host resistance could also explain asymptomatic colonization, as *S. musiva* is known to invade both resistant and susceptible *P. trichocarpa*, but canker expression is suppressed in the former (32, 33, 110). High relative *S. musiva* abundance in some asymptomatic tissues indicates that tissue necrosis and other physiological changes associated with canker development are not required for the pathogenic dominance of the stem mycobiome.

Stem cankering in the absence of *S. musiva* can be partially explained by the inferred presence of canker pathogens *Cytospora* sp. and *Cryptosphaeria pullmanensis* (101, 104, 105, 111, 112). Non-*S*. *musiva* cankering may be the result of bacterial infection, poplar borer beetle (*Saperda calcarata*) damage, or opportunistic infection by typically non-pathogenic fungal endophytes. The inferred absence of *S. musiva* in some cankered tissue samples is congruent with preliminary qPCR assays, where 5 of 13 sampled cankers tested negative for *S. musiva* (69, 113). Similar endophyte studies of common garden *Populus* have also detected the simultaneous presence of multiple potential pathogens (24, 114, 115). Our results provide additional evidence that co-infection is a common phenomenon within diseased *P. trichocarpa* stands, and that multi-pathogen interaction may shape *P. trichocarpa* pathobiome dynamics (50).

### Stem cankering is associated with reduced fungal endophyte diversity at the site of infection

Across pathosystems and plant compartments, filamentous fungal pathogen infection is predominantly associated with reduced endophyte diversity (10). Our results corroborate this pattern. Septoria stem canker expression corresponded with reduced mycobiome diversity at the site of infection, consistent with our hypothesis that symptomatic stem tissues host less diverse fungal communities than asymptomatic tissues. Although turnover between healthy and cankered tissue communities was high, our hypothesis that this pattern is explained by symptom development was not supported, as turnover was uniformly high across all within- and between-disease status comparisons. This relationship could be the result of localized interactions between *S. musiva* and resident fungal endophytes. It could also reflect a connection between pre-infection mycobiome composition and *S. musiva* infection success.

Many hemibiotrophic fungal phytopathogens manipulate resident endophyte communities to facilitate infection (10, 12). Our study does not allow for conclusive inference of the mechanisms by which *S. musiva* may alter the *P. trichocarpa* stem mycobiome during infection. However, *S. musiva*’s tendency to dominate resident fungal endophyte communities at the site of infection suggests localized processes, including microhabitat modification or direct chemical antagonism, may be involved.

Successful plant pathogens commonly manipulate the physiological and biochemical properties of their target microhabitats to gain a competitive advantage (11, 22, 116). These manipulations are often achieved through specialized effector release. For example, various hemibiotrophic bacterial pathogens, including *Pseudomonas syringae* and *Xanthomonas* spp., secrete effector proteins that target and disrupt host abscisic acid osmosignaling to dysregulate stomatal closure and enrich water and nutrients in the leaf apoplast of *Arabidopsis* (117, 118). Effectors secreted by *S. musiva* may similarly facilitate niche modification in vascular stem tissues of *Populus*, enabling resource monopolization and indirectly excluding resident fungal endophytes during biotrophic infection (61). Dramatic microhabitat alteration and niche loss associated with cell wall degradation during necrotrophic infection may further exclude resident endophytes, reinforcing *S. musiva* dominance in symptomatic tissues.

Effectors secreted by *S. musiva* may also directly antagonize resident microbiota. Selective suppression of competitive or antagonistic endophytes through the release of antimicrobial effectors appears to be a highly adaptive trait of many plant pathogens (12). For example, the hemibiotrophic fungus *Verticillium dahliae* produces well-characterized antimicrobial effectors that selectively suppress antagonistic endophytes of cotton and tomato (119–121). While the utilization of antimycotic effectors by *S. musiva* remains hypothetical, integrated biochemical and culture-based analyses of *S. musiva*-endophyte interactions could mechanistically substantiate these observations and advance our understanding of *S. musiva*’s diverse effector repertoire (40).

Pre-infection mycobiome composition may also explain the observed association between fungal endophyte diversity and canker expression. Manipulative experiments repeatedly identify biodiversity as a major determinant of invasion resistance in soil and plant-associated microbial communities, whereby low-diversity communities are more susceptible to invasion than high-diversity communities (61, 65, 122). This relationship is particularly relevant for systems where the invader and resident microbiota compete for similar resources, as has been documented in the Dutch elm disease pathosystem (61, 64, 109). Similarly, competitive relationships may be common across hardwood stem pathosystems due to the unique spatial and nutrient constraints of the vascular microhabitat (19, 23). Spatial or genotype-associated variation of stem mycobiome diversity within the *P. trichocarpa* common garden may have meaningfully shaped pre-infestation invasion resistance, producing differential susceptibility to *S. musiva* colonization. If so, *S. musiva* establishment and canker expression may have occurred more frequently in hosts with reduced pre-infection mycobiome diversity.

Disentangling the causal relationship between Septoria stem canker expression and mycobiome diversity is not possible with our data. However, considerable evidence from similar pathosystems supports both pathogen-induced phytobiome manipulation and phytobiome-mediated modulation of infection success as plausible explanations. Dedicated manipulative experimentation will be necessary to determine the contribution of each of these processes to disease development and phytobiome dynamics in the Septoria stem canker pathosystem.

Our hypothesis that asymptomatic stem tissues of cankered trees harbor less diverse and compositionally distinct fungal endophyte communities compared with those of entirely healthy stems, potentially reflecting relative abundance variation and taxa loss driven by systemic, host-mediated, infection-related physiological changes, was not supported by our results. While various lines of evidence suggest systemic immune response can alter endophyte composition across plant compartments, this study found no evidence of mycobiome modification in asymptomatic tissues of infected stems (10, 48, 123). Our results are partially congruent with those of foliar *Populus* endophyte studies (124). For example, a similar study of common garden *P. trichocarpa* and *P. deltoides* found foliar bacterial and fungal endophyte community response to *S. musiva* infection was independent of canker expression and non-systemic, though *S. musiva* abundance was positively correlated with alpha diversity in this context (110). It is unclear whether this incongruence arises due to compartment-specific effects or discrepancies between qPCR and ITS amplicon-based estimates of *S. musiva* abundance.

### High-abundance fungal endophytes are positively inter-correlated and negatively correlated with *S. musiva* and stem cankering

Strong correlations among ASVs classified as *Aureobasidium pullulans*, *Filobasidium floriforme*, *Dioszegia* sp., *Buckleyzyma aurantiaca*, and *Buckleyzyma* sp. suggest these taxa may represent a recurrent, highly associated assemblage within the *P. trichocarpa* stem mycobiome. Speculation about the ecological basis of these associations is difficult, as mechanisms of microbial cooperation among these and other endophytes remain poorly characterized. *Aureobasidium pullulans* is a widely described generalist endophyte with a recognized capacity to influence microbial community structure in many plant-associated microbiomes and agricultural pathosystems (125). *Dioszegia* has been described as an important hub taxon in the leaf episphere microbiome of *Arabidopsis thaliana. Dioszegia* was hypothesized to be a keystone epiphyte in this context, as its high network connectivity indicated disproportionate importance for determining microbial assembly and community structure. Additionally, *Dioszegia* was indicated to be functionally redundant with the pathogenic oomycete *Albugo* in manipulation of bacterial community composition, suggesting that *Dioszegia*’s influence on phytobiome structure could meaningfully shape inter-kingdom microbial interactions and potentially alter disease dynamics in *Arabidopsis thaliana* (126–128). In the *P. trichocarpa* stem mycobiome network, a *Dioszegia* sp. ASV held a highly connected position despite low relative abundance, supporting *Dioszegia* as potentially important interactors within the *P. trichocarpa* stem phytobiome. *Filobasidium* and *Buckleyzyma* are commonly reported as endophytes, but the ecological roles of *Filobasidium floriforme* and *Buckleyzyma aurantiaca* remain unclear, making their associations with *Aureobasidium pullulans* and *Dioszegia* sp. particularly noteworthy (106, 129).

As hypothesized, common resident endophytes of the *P. trichocarpa* stem mycobiome are negatively associated with *S. musiva* and Septoria canker expression. The negative correlations between endophytes *Aureobasidium pullulans*, *Filobasidium floriforme*, and *Dothiora* sp. and *S. musiva* may reflect chemical or competitive antagonism, though the directionality of these relationships is unclear. *Aureobasidium pullulans* demonstrates antagonistic activity against a range of plant pathogens, including *Botrytis cinerea* in tomato, *Fusarium culmorum* in wheat, *Rhizoctonia solani* in legumes, and *Ophiostoma novo-ulmi* in elm (109, 130–132). Enzyme and antimicrobial metabolite secretion, as well as competitive exclusion of pathogens, are reported mechanisms of microbial antagonism employed by *Aureobasidium pullulans* (125).

Reciprocally, *S. musiva* may specifically antagonize *Aureobasidium pullulans* during infection. This interaction could be particularly important for mycobiome dynamics in this pathosystem, as selective antagonization of a putatively important interactor like *Aureobasidium pullulans* could disproportionately alter stem mycobiome ecology. A similar dynamic has been reported in the phyllosphere mycobiome of chili pepper affected by *Fusarium* wilt, where selective regulation of key fungal endophytes was shown to destabilize microecological network structure (26). The directionality of these *S. musiva*-endophyte relationships and their potential underlying ecological mechanisms remain unclear. However, our results suggest that *S. musiva* may antagonize or competitively exclude specific fungal endophytes to facilitate infection. Alternatively, key fungal endophytes may antagonize or competitively exclude *S. musiva* during colonization, meaning their presence in the pre-infection mycobiome would reduce invasion success.

Microecological conclusions drawn from metabarcoding data must be tempered with discussion of their analytical limitations. The effects of inherent compositional and sequence-depth biases on diversity and correlation analyses are widely described (133, 134). Careful consideration was taken to computationally control these biases through employment of repeated empirical sequence count rarefaction, a compositionally appropriate beta-dissimilarity metric (Aitchison distance), and a specialized correlation estimation method (SparCC). However, indirect effects resulting from the compositional nature of these data may still constrain interpretation (135, 136). For example, negative correlations between high-abundance fungal endophytes and *S. musiva* could reflect antagonism or competitive exclusion, whereby *S. musiva* proliferation reduces the absolute abundances of resident fungal endophytes (or vice versa). However, this pattern could also be a compositional artifact, whereby the relative abundances of resident fungal endophytes are artificially reduced as *S. musiva* proliferates without meaningful exclusion or antagonism (or vice versa). Further investigation is required to assess the extent to which compositional biases, potentially exacerbated by high host amplification, have influenced the mycobiome–disease dynamics observed in this study.

The microecological inferences discussed here strongly reflect processes described in similar plant pathosystems (10, 12). However, robust metabolic, genomic, and ecological evidence will be necessary to understand how *S. musiva* may or may not alter the vascular stem mycobiome during infection. Additionally, taxonomic classification of ITS2 ASVs may be affected by reference bias or misclassification. While this uncertainty does not affect conclusions related to the community-level dynamics of this pathosystem, the presence and potential interaction of inferred taxa should remain hypothetical unless supported by additional culture-based or molecular evidence. Additionally, temporal pathogen–mycobiome dynamics, including variation in canker age, were not captured in this study and may confound the observed patterns. Future work incorporating longitudinal sampling of controlled, inoculated cankers at multiple time points could resolve patterns of mycobiome succession across canker development stages. In addition, future studies characterizing bacterial community dynamics and biochemical variation across symptomatic and asymptomatic *Populus* stem tissues will provide a more complete understanding of microecological processes in this pathosystem.

### Conclusion

With growing recognition of the phytobiome as a dynamic and complex component of plant systems, understanding its functional role in disease processes has become an important research objective. In this study, we investigated the understudied vascular stem mycobiome of the model hardwood species *P. trichocarpa* under *S. musiva* disease pressure. A clear association between fungal endophyte diversity and Septoria canker expression suggests that *S. musiva* may locally modify the vascular stem mycobiome. This pattern could also reflect a relationship between pre-infection mycobiome composition and *S. musiva* infection success, potentially underpinned by host-genotype or biogeographic factors. Further research efforts are necessary to clarify how these processes may individually or interactively contribute to disease outcomes in the Septoria stem canker pathosystem. We also identified fungal endophytes associated with *S. musiva* and canker expression, suggesting their potential ecological importance in this diseased context. Future manipulative experimentation could illuminate how these key fungal endophytes individually interact with *S. musiva* or collectively modulate the invasion resistance of the vascular stem phytobiome. Our findings advance understanding of the vascular *P. trichocarpa* stem mycobiome and its ecological organization under *S. musiva* disease pressure, informing efforts to improve integrative disease management strategies and predictive disease modeling in this and related hardwood stem pathosystems.

## Data Availability

Raw sequence data are publicly available in the National Center for Biotechnology Information Sequence Read Archive under BioProject accession number PRJNA1365169. Additional data and analysis code are available on GitHub: https://github.com/LeBoldus-Lab/poplar-stem-mycobiome.

## SUPPLEMENTAL MATERIAL

Figure S1 msystems.00055-26-s0001.pdf Fungal endophyte community alpha diversity.

Figure S2 msystems.00055-26-s0002.pdf Total Jaccard dissimilarity and the turnover component of Jaccard dissimilarity among fungal endophyte communities.

Legends msystems.00055-26-s0003.docx Supplemental figure legends.

ASM does not own the copyrights to Supplemental Material that may be linked to, or accessed through, an article. The authors have granted ASM a non-exclusive, world-wide license to publish the Supplemental Material files. Please contact the corresponding author directly for reuse.
