# Supplementary figures and images for: The interaction between Septoria stem canker and the mycobiome of Populus trichocarpa stems
Source: mSystems. 2026 Jun 15;11(7):e00055-26. doi: 10.1128/msystems.00055-26 (PMC13387005; doi:10.1128/msystems.00055-26)

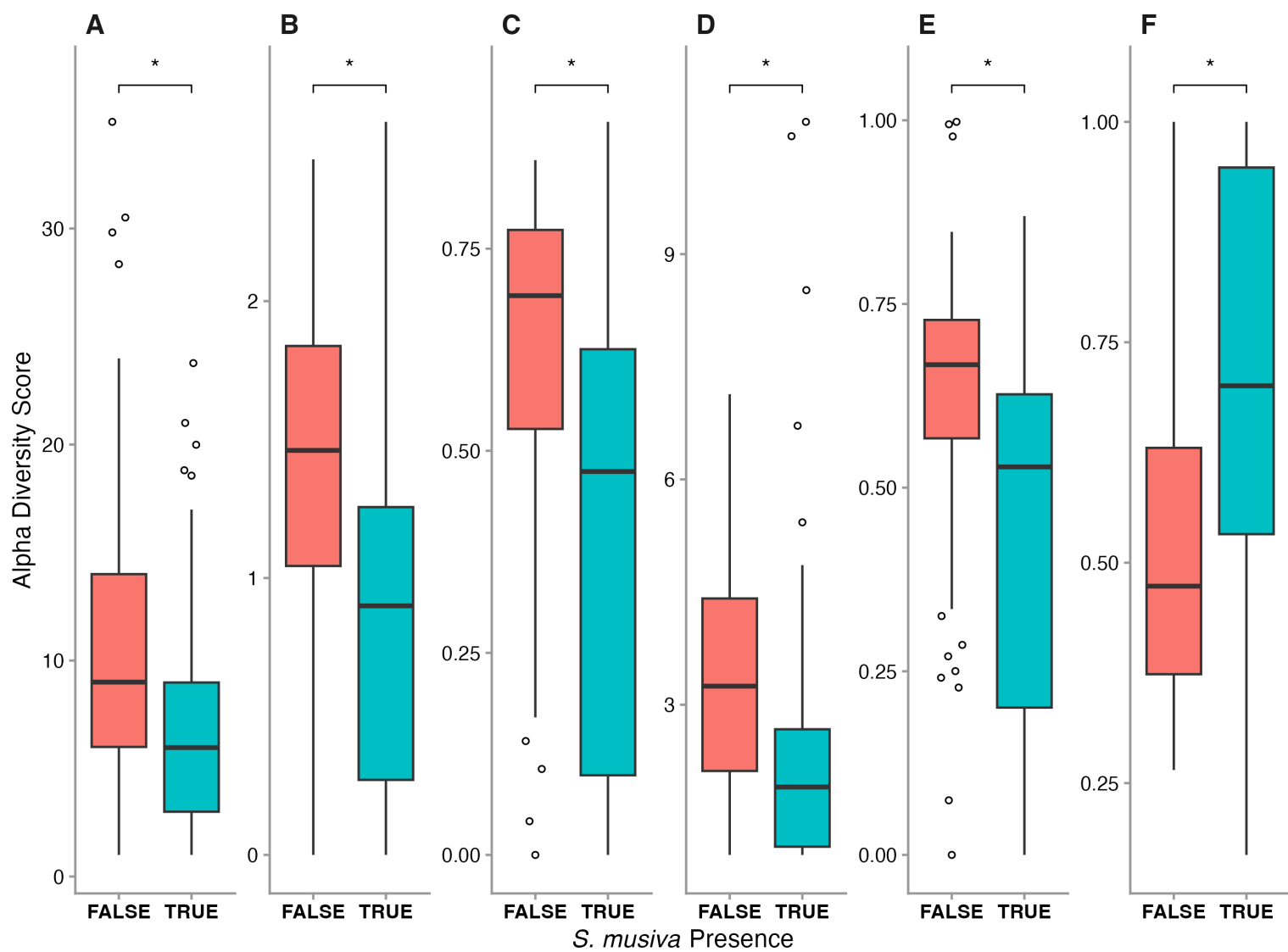

Supplement: Figure S1 — Fungal endophyte community alpha diversity. [file msystems.00055-26-s0001.pdf]

**A**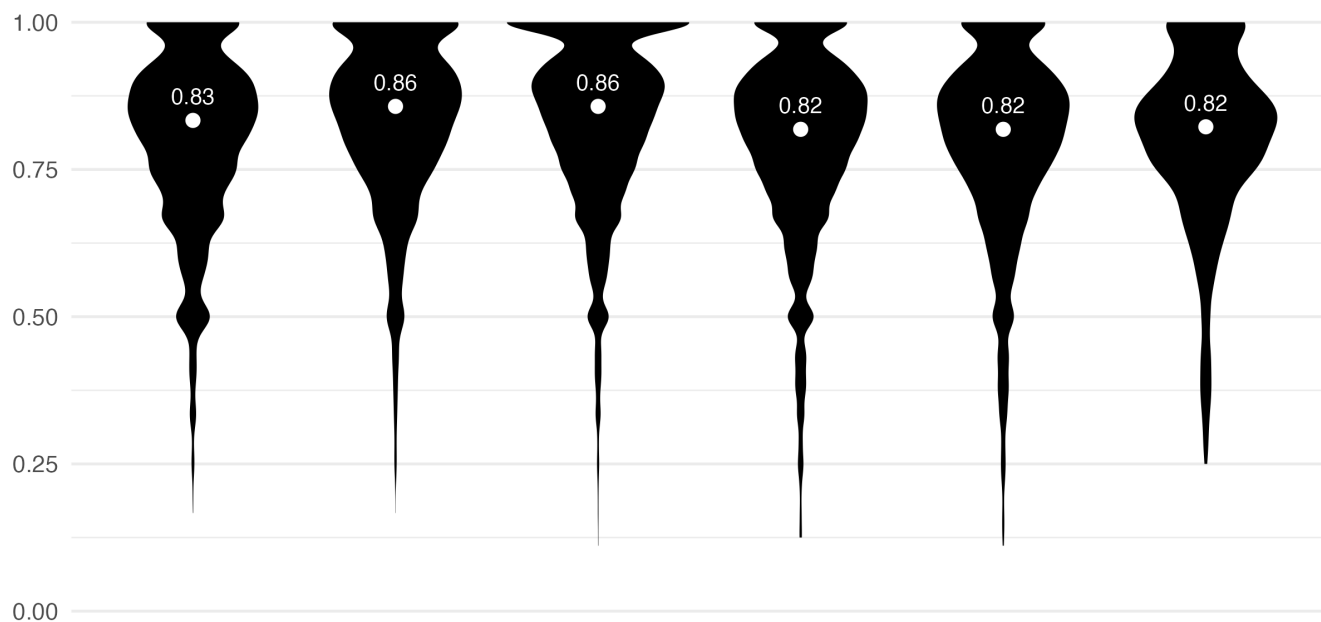**B**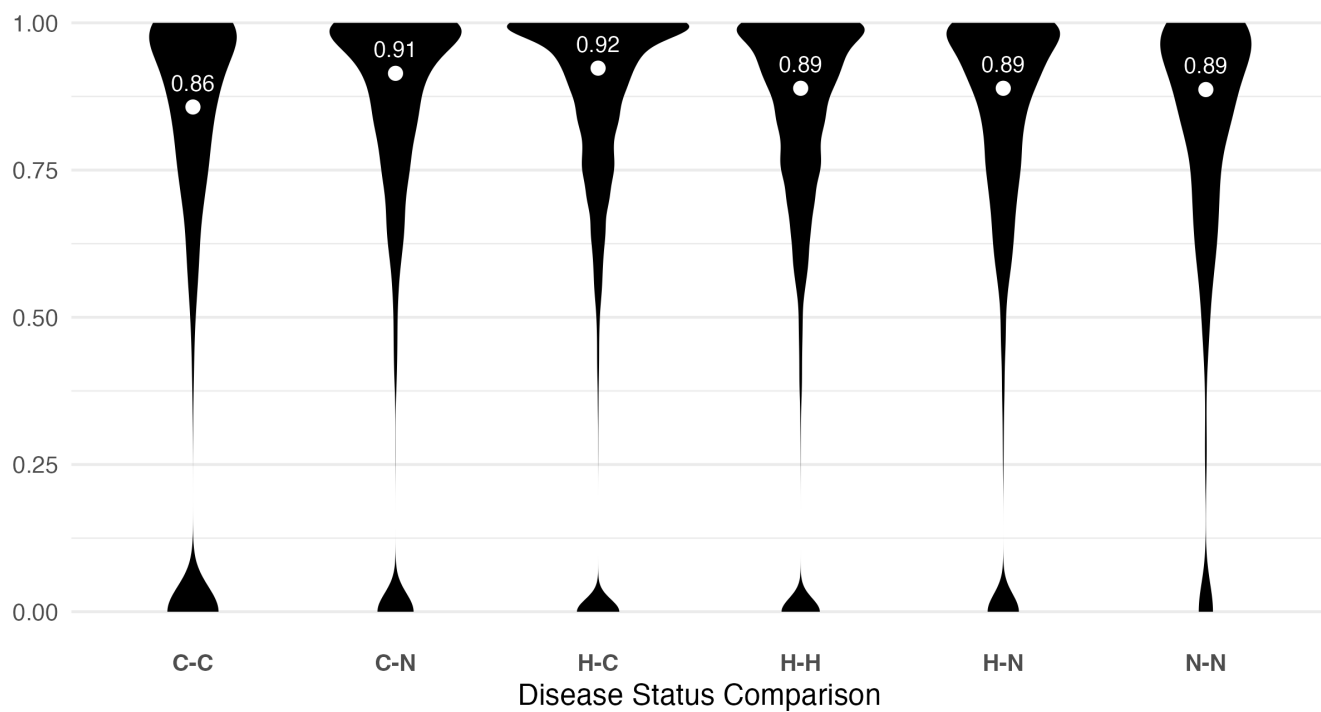

Supplement: Figure S2 — Total Jaccard dissimilarity and the turnover component of Jaccard dissimilarity among fungal endophyte communities. [file msystems.00055-26-s0002.pdf]
