# Supplementary material for: The interaction between Septoria stem canker and the mycobiome of Populus trichocarpa stems
Source: mSystems. 2026 Jun 15;11(7):e00055-26. doi: 10.1128/msystems.00055-26 (PMC13387005; doi:10.1128/msystems.00055-26)
Supplement: Legends — Supplemental figure legends. [file msystems.00055-26-s0003.docx]

**Supplementary Figure Captions**

**Figure S1.** Box and whisker plots depicting fungal endophyte community alpha diversity grouped by *Sphaerulina musiva* ASV presence–absence. Samples from all disease status groups (healthy, n = 103; cankered, n = 62; and non-cankered, n = 20) are included. Five different indices were compared: richness (A), Shannon-Weaver (B), Simpson (C), Inverse Simpson (D), Pielou evenness (E), and Berger-Parker dominance (F). Mean diversity scores were calculated for each sample after repeated rarefaction. Asterisks denote statistically significant differences in mean values between groups, determined by Welch’s two-sample *t*-tests (*P* < 0.05).

**Figure S2.** Violin plots depicting (A) total Jaccard dissimilarity and (B) the turnover component of Jaccard dissimilarity among fungal endophyte communities, grouped by pairwise disease status comparisons among samples from healthy (H; n = 103), cankered (C; n = 62), and non-cankered (N; n = 20) tissues. White dots indicate median values and are labeled with their corresponding values.
